# Neural Control of Dynamic 3-Dimensional Skin Papillae for Cuttlefish Camouflage

**DOI:** 10.1016/j.isci.2018.01.001

**Published:** 2018-01-31

**Authors:** Paloma T. Gonzalez-Bellido, Alexia T. Scaros, Roger T. Hanlon, Trevor J. Wardill

**Affiliations:** 1 Marine Biological Laboratory, MBL Street, Woods Hole, MA 02543-1015, USA; 2 Department of Physiology, Development and Neuroscience, University of Cambridge, Downing Place, Cambridge CB2 3EG, UK; 3 Department of Physiology and Biophysics, Dalhousie University, College Street, Halifax, NS B3H 4R2, Canada

**Keywords:** Animal Physiology, Neuroanatomy, Evolutionary Biology

## Abstract

The color and pattern changing abilities of octopus, squid, and cuttlefish via chromatophore neuro-muscular organs are unparalleled. Cuttlefish and octopuses also have a unique muscular hydrostat system in their skin. When this system is expressed, dermal bumps called papillae disrupt body shape and imitate the fine texture of surrounding objects, yet the control system is unknown. Here we report for papillae: (1) the motoneurons and the neurotransmitters that control activation and relaxation, (2) a physiologically fast expression and retraction system, and (3) a complex of smooth and striated muscles that enables long-term expression of papillae through sustained tension in the absence of neural input. The neural circuits controlling acute shape-shifting skin papillae in cuttlefish show homology to the iridescence circuits in squids. The sustained tension in papillary muscles for long-term camouflage utilizes muscle heterogeneity and points toward the existence of a “catch-like” mechanism that would reduce the necessary energy expenditure.

## Highlights

• Papillae shape and skin tone motoneurons are located in the stellate ganglion
• Papillae exhibit extended “catch-like” tension in the absence of neural stimulation
• FMRFa, ACh, and L-Glut activate and 5-HT relaxes papillae/skin tension
• Sepia skin is composed of distinct muscle layers with striated and smooth muscles

## Graphical Abstract

**Figure:**
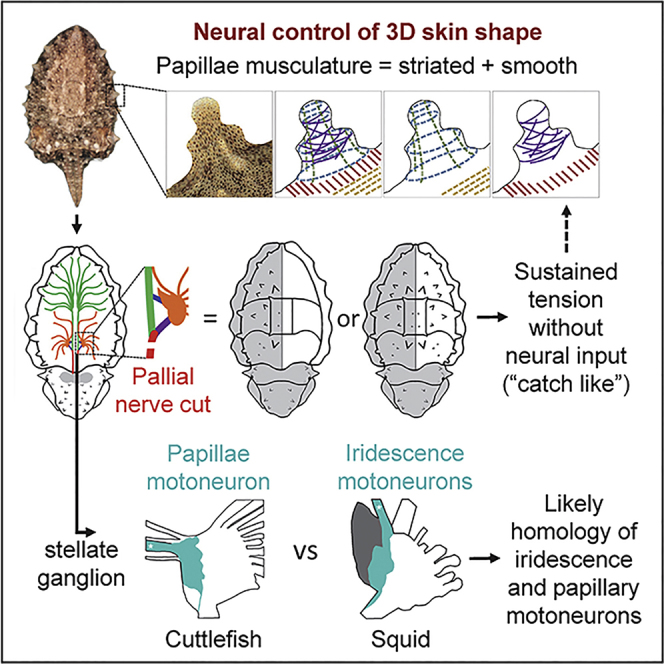

## Introduction

**Figure 1:**
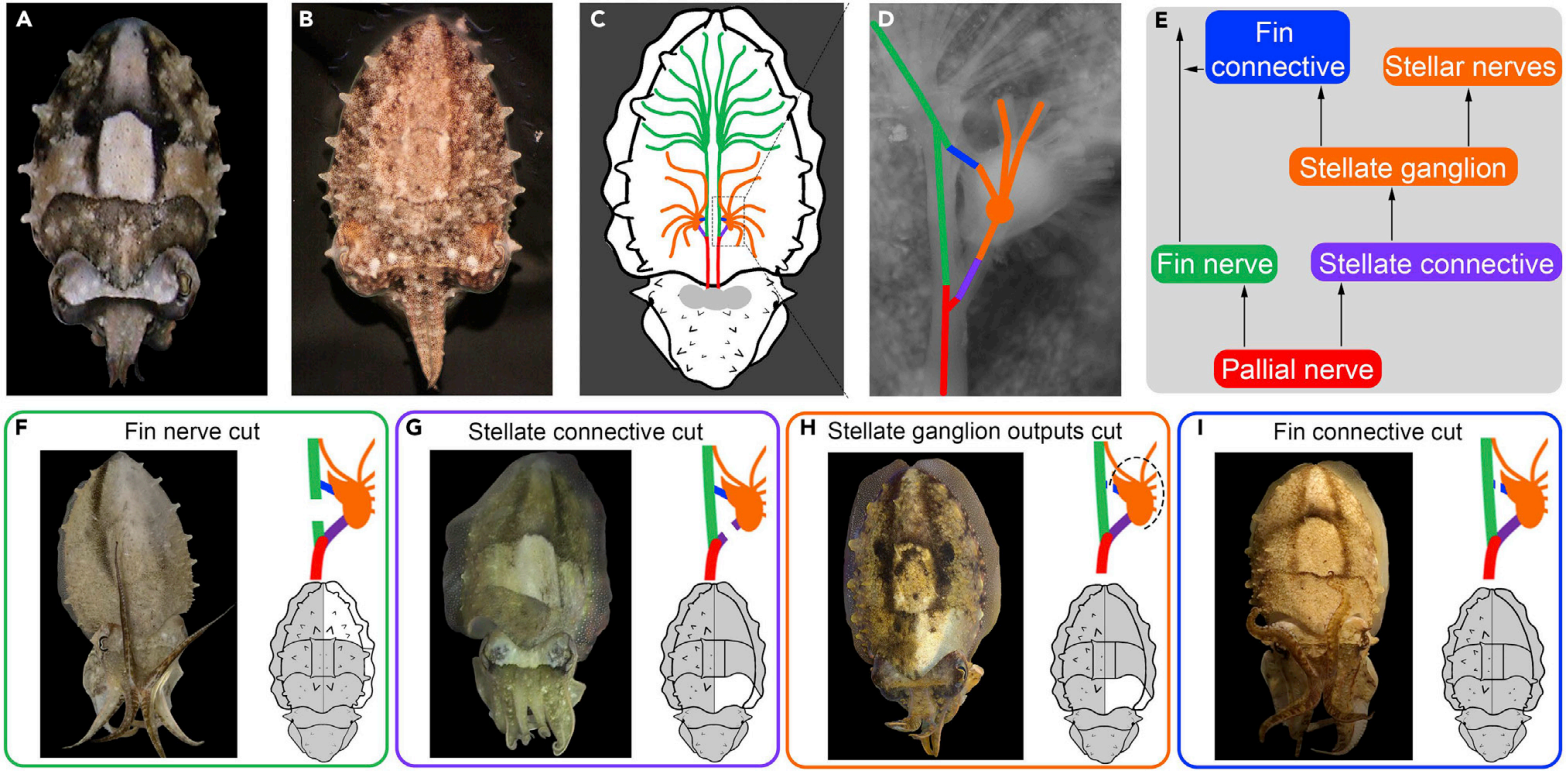
Neural Control System of the Cuttlefish Mantle and Fins (A) An adult cuttlefish, *Sepia officinalis,* showing disrupted camouflage pattern while expressing papillae. (B) A juvenile cuttlefish with many erect papillae over its dorsal surface. (C) The peripheral nervous system for mantle, skin, and fins; the pallial nerve (red) splits into the stellate connective (purple) and the fin nerve (green). The stellate connective travels into the stellate ganglion (orange), whereas the fin nerve projects directly to the fin. The most medial stellate nerves project out of the ganglion and rejoin the fin nerve via the fin connective (blue). Note that branching of the fin nerve, after the fin connective, happens close to the stellate ganglion as shown in D but has been separated in C for diagrammatic purposes. (D) The main peripheral nerves and connectives overlaid on an image of a dissected *S. officinalis* stellate ganglion. (E) A diagrammatic representation of the cuttlefish peripheral nervous system shown in D. (F) Phenotype resulting from severing the left fin nerve between the pallial and fin connective. (G) Phenotype resulting from severing the left stellate connective. (H) Phenotype resulting from severing all visible left stellate outputs (along dashed line). (I) Phenotype resulting from severing the left fin connective. See also Figure S1 and Movie S1.

Coleoid cephalopods (squid, cuttlefish, and octopus) internalized their shell more than 400 Mya (Tanner et al., 2017). Their soft bodies not only provided a unique set of opportunities related to motor control but also rendered them more susceptible to predation. Their solutions are unique in the animal kingdom. For example, their skin is covered with chromatophores, neurally innervated muscular organs of different colors (Messenger, 2001). The chromatophore motoneurons are located in the brain, and their activation allows the animal to quickly and dynamically change color and pattern (Messenger, 2001, Young, 1972). In addition, squids have patches of skin cells called iridophores, each of which contains reflectin, a protein that shifts conformation to reflect light when the cells are exposed to acetylcholine (ACh) (Hanlon et al., 1990), thus yielding bright specific hues across the visible spectrum, including short-wavelength colors, such as blue and green, which are not produced by the pigmented chromatophores (Izumi et al., 2010). Recently, the iridescence system was shown to be neurally controlled, but unlike those of the chromatophores, their motoneurons reside in the stellate ganglion (Gonzalez-Bellido et al., 2014). Because most cephalopods have been shown to be color blind (e.g., Marshall and Messenger, 1996, Mäthger et al., 2006, but also see Stubbs and Stubbs, 2016), it is currently thought that the highly polarized light reflected from activated iridophores is used as a signal for intraspecific communication (Mäthger et al., 2009). However, the origin and purpose of this system still remain to be elucidated. Cuttlefish and octopus possess yet another exquisite adaptation; within their skin, muscle groups work in an antagonistic and agonistic manner, forming miniature muscular hydrostats. When activated, these muscles create dermal bumps called papillae (Figures 1A and 1B). Individual papilla in the living cuttlefish can be fully expanded (or retracted) in less than 1 s (Panetta et al., 2017). Different levels of structural complexity lead to larger and more intricate papillary shapes, with each species having a fixed repertoire of papillae shapes (Allen et al., 2013, Allen et al., 2014). This ability allows these benthic animals to be cryptic on substrates or masquerade as nearby objects, such as kelp, algae, or coral (Panetta et al., 2017), but the neural circuit and muscular anatomy that underlie it are unknown to date.

## Results

### Neural Control of Papillae Is Routed through the Stellate Ganglion and Exits through a Nerve Equivalent to the Squid Fin Iridescence Nerve

In addition to papillae, cuttlefish skin is covered in chromatophore organs comprising a cytoelastic sac of pigment expanded by a radial array of muscles. Both of these skin elements are visually driven (Panetta et al., 2017) yet controlled by different circuits, as papillae and chromatophores can be activated independently (Figures 1A and 1B). The motoneurons that drive the chromatophore musculature originate in the brain lobes and descend through the pallial nerve (Young, 1972, Messenger, 2001), which splits into the fin nerve and the stellate connective (Figures 1C–1E). The fin nerve provides an ideal substrate to test if the papillae motoneurons project parallel to the chromatophore motoneurons because (1) it is relatively easy to access in live anesthetized animals and (2) chromatophore motoneurons that target the posterior part of the mantle and the fins travel directly through the fin nerve (Gaston and Tublitz, 2004). Thus, we severed the fin nerve *in vivo* under anesthesia, and upon recovery we noted that the fin was paralyzed and chromatophore activity had ceased from the posterior mantle tip to the posterior bar, but papillae expression was not affected (Figure 1F; n = 2; Movie S1A). In the control surgery, we cut the stellate connective, which yielded the inverse phenotype: chromatophores blanched from the anterior end to the location of the anterior bar, but papillae control was entirely lost ipsilateral to the cut (Figure 1G; n = 2; Movies S1B and S1C). The same phenotype was obtained when all the outputs of the stellate ganglion were severed (Figure 1H; n = 1; Figure S1). We further confirmed the results by electrically stimulating the stumps of the fin nerve and the stellate connective in *ex vivo* skin preparations (Figures S2A–S2D; n = 2). Note that all procedures carried out in this study comply with institutional recommendations for cephalopods and follow the tenants outlined by the Animal Welfare Act.

**Figure 2:**
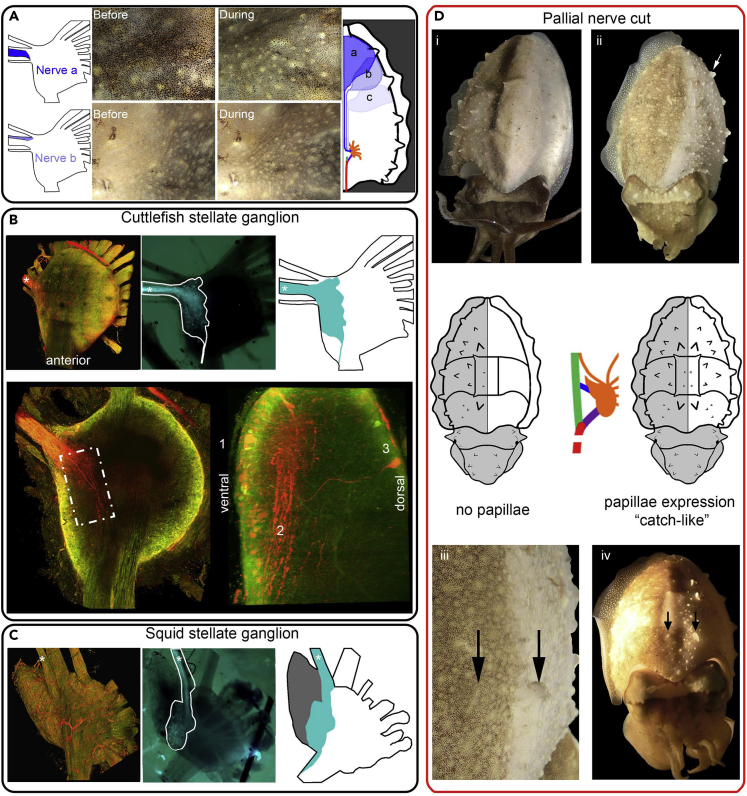
Nerve Labeling and Denervation of Papillae Control System (A) Effect of the electrical stimulation of two of the nerves forming the fin connective and diagrammatic representation of the areas that they innervate. (B) Images of backfilled *S. officinalis* fin connective nerve (indicated by *) showing three populations of neurons: (1) formed by the majority of the fibers labeled, with the cells bodies located in the ventral medial wall of the stellate ganglion; (2) a small number of cell bodies in the dorsal-medial wall; and (3) a small number of fibers that continue through into the pallial nerve. Dashed box indicates volume used for the maximum intensity projection image shown on the right. (C) The cell bodies labeled by backfilling the squid fin connective (indicated by *) are found in a similar location (see first report in [Gonzalez-Bellido et al., 2014]). (D) (i) Phenotype resulting from severing the pallial nerve under anesthesia when papillae were not being expressed. (ii) Phenotype resulting from severing the pallial nerve under anesthesia when papillae were expressed (example indicated by white arrow on lateral papillae). (iii and iv) Higher magnification view of papillae in a catch-like state (right) vs unexpressed pair (left) (indicated by black arrows). Results were all seen instantly after severing the nerve, and prolonged thereafter. See also Figure S2.

The above results confirm that the neural activity driving the mantle papillae is routed through the stellate ganglion. This situation is similar to the neural circuit for controlling squid iridescence. In squid, the motoneurons for iridescence originate in the stellate ganglion, then exit through a medial/posterior stellar nerve (named the fin iridescence nerve), and then join the fin nerve (Gonzalez-Bellido et al., 2014). Upon de-sheathing, we found that cuttlefish also possess a neural connection between the stellate ganglion and the fin nerve, hereafter referred to as the fin connective (Figures 1C–1E). Severing the fin connective abolished the animal's ability to express papillae ipsilateral to the cut, but fin and chromatophore control remained intact (Figure 1I; n = 1; Movie S1D). We also carried out the reverse experiment and stimulated with suction electrodes three of the nerves that make up the fin connective. This resulted in papillae expression and skin bunching in a topographic manner (Figure 2A; n = 2; Movie S2A). During stimulation the skin also blanched because of chromatophore relaxation (Movie S2A); this may be a passive process resulting from skin bunching or an active process driven by inhibitory neurons. By loading a fin connective nerve with Lucifer yellow and by imaging the entire ganglia with a two-photon microscope, we found that the majority of the cell bodies are located in the medial-ventral wall of the stellate ganglion (Figure 2B; n = 3; Movie S2B). Our anatomical and functional findings match those of the motoneurons that form the squid fin iridescence nerve (Gonzalez-Bellido et al., 2014), including the presence of a minor number of cell bodies in the dorsal wall and a few fibers that continue and join the pallial nerve (Figure 2C). The implications of these findings with regard to the evolution of dynamic control of iridescence and 3-dimensional (D) skin texture are considered in the discussion.

### Pallial Nerve Denervation and Sustained Papillae Expression

As a final control to the aforementioned set of experiments, we also severed the pallial nerve (Figure 2D; n = 2). As the pallial nerves carry all neural signals between the brain and the mantle and fins, we expected this cut to result in complete ipsilateral paralysis. As anticipated, in the first animal, (1) the fin became paralyzed and passively curled under the body, (2) the skin drooped, which shifted the skin's midline toward the paralyzed side, and (3) the chromatophores were instantly retracted, which produced the observed skin blanching leaving the cuttlebone visible (Figure 2Di; Movie S2C). However, on the second animal, we found that after recovery from anesthesia, the papillae remained completely expressed for over an hour. This was true even for the large lateral multi-lobed papillae that require a significant level of coordinated motor control (Figure 2Dii, white arrows). To encourage the animal to relax the papillae and to test if other behaviors were unaffected, we placed this animal on different substrates. This treatment elicited the expected changes in chromatophore and papillae expression on the control side, but the denervated side remained pale with fully expressed papillae (Figure 2Diii-iv; see also Movie S1C where a stellate connective cut left fully expressed papillae). Tetanic post-severing activation is very unlikely to be responsible for the observed phenotype for the following reasons: (1) *Sepia* skin functions as a muscular hydrostat. To maintain the normal papillae form observed, tetanic post-severing activation would have to result in a precise spatial and temporal pattern of muscle recruitment. (2) Tetanic post-severing activation would lead to indiscriminate activation of the axons in such connective (although our electrophysiological experiments demonstrate that such activation leads to skin bunching and papillae expression, the shape is not like that seen in alive animals, neither controls nor those with stuck papillae phenotype; Figures 1, 2A, and 2D; Movies S1C and S2A). (3) Tetanic post-severing activation would also affect other axons, but we have never seen chromatophores stay expanded after severing the pallial nerve. A feasible alternative is that at the time of the nerve dissection, papillary muscles were in a state that allows long-term maintenance of tone through slow relaxation, akin to that achieved by the smooth muscles of vertebrates through a “latch-bridge” mechanism (Dillon et al., 1981) or the smooth muscles of bivalve mollusks through a “catch” mechanism (Twarog, 1976). Catch muscles have been well studied in bivalves (Andruchov et al., 2006, Andruchova et al., 2005, Ishii et al., 1986), a molluscan lineage, but have not been reported in cephalopods. Such catch-like ability may have evolved independently in cephalopods and may differ substantially from that of bivalves. Next, we investigated which neurotransmitters control papillae expression, as these may provide clues about their mechanism of action.

### Neuropeptide and Neuromodulators Controlling Papillae Expression

**Figure 3:**
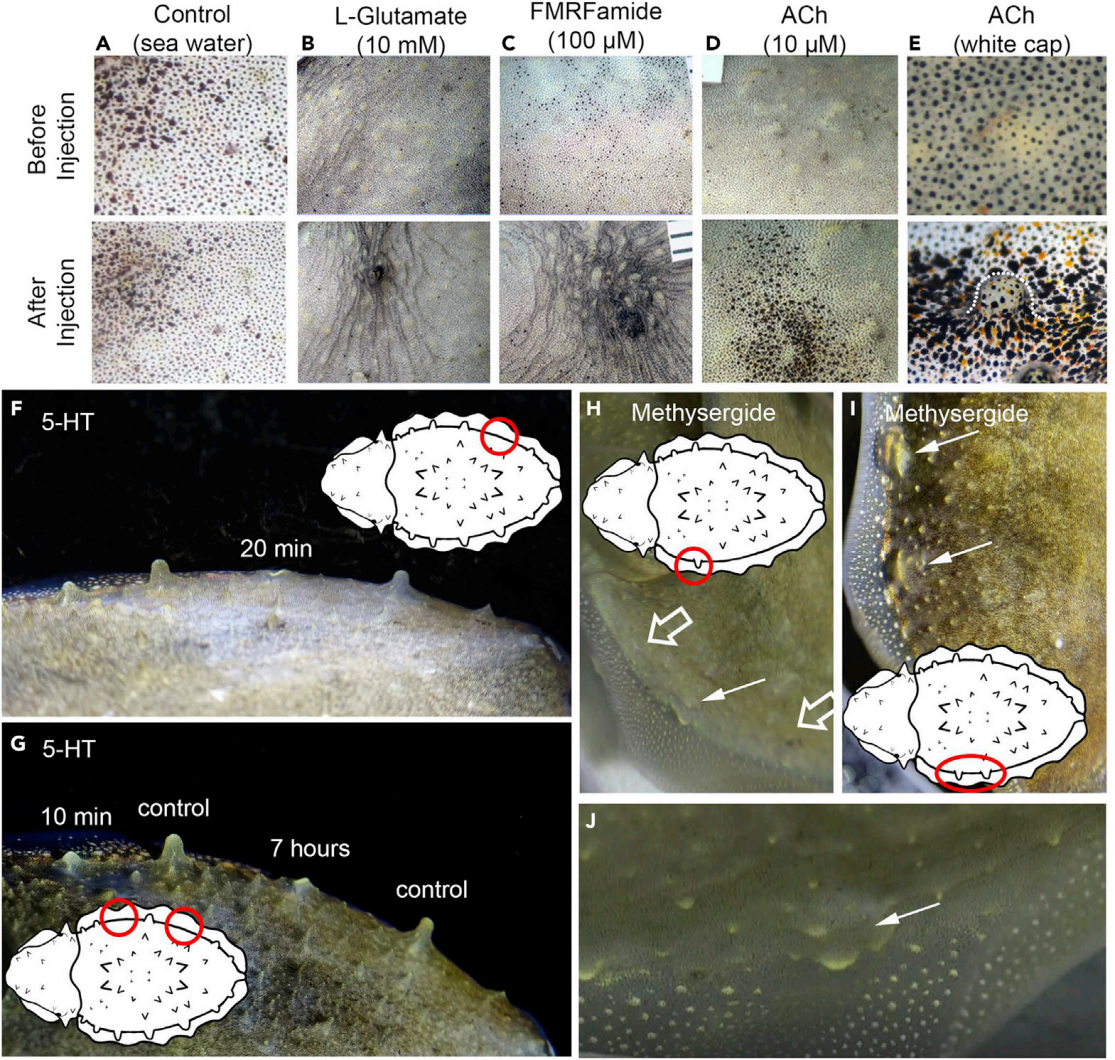
Pharmacological Stimulation and Inhibition of Papillae (A) Small dorsal mantle papillae in *ex vivo* mantle preparation before injection and 2.5 min after injection of seawater. (B) Small dorsal mantle papillae *ex vivo* before injection of 10 mM L-glutamate and 2.5 min after injection. (C) Small dorsal mantle papillae *ex vivo* before injection of 100 μM FMRFamide and 2.5 min after injection. (D) Small dorsal mantle papillae *ex vivo* before injection of 10 mM acetylcholine (ACh) and 2.5 min after injection. (E) Higher magnification of small dorsal mantle papilla in a control preparation and a different small dorsal papilla after acetylcholine (ACh) injection. Expressed papillae shape outlined by a dashed line. (F) Major lateral mantle papillae shown 20 min after *in vivo* subcutaneous injection of serotonin (5-HT, 1 mM) with adjacent control papillae. Red circles here and below indicate injected papillae. (G) The same major lateral mantle papillae shown in F, here 7 hr post injection, and a second serotonin injection in neighboring papilla 10 min prior. (H) Major lateral mantle papillae treated with methysergide injection (10% dilution of 28.29 mM); solid arrow indicates affected papillae, empty arrows indicate neighboring unaffected unexpressed papillae. (I) Two papillae injected with 1% methysergide. Solid arrows, treatment; open arrow, control papillae. (J) Higher magnification of skin near methysergide injection (10% dilution of 28.29 mM). See also Figure S3 and Movie S3.

We selected the neurotransmitters/neuromodulators to be tested by their role in the catch state of bivalve muscles or on cephalopod chromatophore activation. They were diluted in seawater and injected locally and subcutaneously in the most basal layers of the skin. The injection site was easily recognized, as the injected fluid created a bolus that dissipated slowly. Control injections of sea water did not produce a response (*ex vivo* Figure 3A; *in vivo* Figure S3). To determine the functional concentration, compounds were tested at various concentrations (see Table S1). The lowest concentration that produced a reproducible response is reported below.

We first tested compounds with known excitatory roles: L-glutamate ((2S)-2-aminopentanedioic acid), FMRFamide (H-Phe-Met-Arg-Phe-NH2), and acetylcholine (2-acetyloxyethyl(trimethyl)azanium). L-Glutamate produces fast chromatophore expansion in all tested cephalopod species (Florey et al., 1985, Messenger, 2001, Di Cosmo et al., 2006). As expected, L-glutamate caused chromatophore expansion (1 mM; n = 2), but it also elicited skin buckling and expression of small dorsal papillae (10 mM; n = 4; Figure 3B). Thus, L-glutamate appears to be a generalized fast excitatory neurotransmitter in skin muscles, including papillae. In contrast to L-glutamate, FMRFamide affects only octopus and cuttlefish chromatophores, and the response is slower (Loi and Tublitz, 2000, Messenger, 2001). FMRFamide is known to elicit catch state in bivalve muscle (Painter, 1982), and at a lower concentration, FMRFamide was sufficient to elicit strong chromatophore expansion, skin buckling, and expression of papillae (100 μM; n = 9; Figure 3C); even 10 μM FMRFamide elicited a minor skin bunching response (n = 1; Table S1). Because acetylcholine can also induce catch in bivalve muscles, we also tested this compound. Just like FMRFamide, acetylcholine produced a slow but constant onset (several seconds) of chromatophore expansion, and the leucophore layer that sits at the tip of the small papillae took a spherical appearance (10 μM, n = 1; Figure 3D). We refer to this phenomenon as “white caps” (Figure 3E). Although a higher concentration was necessary (100 μM), acetylcholine also induced skin bunching (n = 2; Table S1).

The results thus far pointed to FMRFamide, acetylcholine, and L-glutamate as the main papillae neurotransmitters. As serotonin (3-(2-aminoethyl)-1H-indol-5-ol; 5-HT) is known to release the catch in bivalves, we injected 5-HT *in vivo* to make sure that the papillae were being expressed naturally by the animal. Serotonin (1 mM, n = 4) suppressed excitation to the point where no papillary bump was visible near the site of injection, even when all other papillae around it were fully expressed (Figures 3F and 3G). The effect was clearly visible within 10 minutes of the injection, complete abolition continued for over 20 minutes, and papillary expression was still not completely recovered 7 hours later. As a control, we injected methysergide ((6aR,9R)-N-[(2S)-1-hydroxybutan-2-yl]-4,7-dimethyl-6,6a,8,9-tetrahydroindolo[4,3-fg]quinoline-9-carboxamide;(Z)-but-2-enedioic acid; undiluted and diluted 50%, 10%, and 1% of methysergide maleate salt, 28.29 mM; n = 13), a strong inhibitor of the 5-HT pathway. Methysergide injection left expressed papillae in a catch-like state, as they were left in the expressed position for over an hour, even when the rest of the animal was not expressing any papillae (10%; Figure 3H; n = 3; 1%; Figure 3I; n = 5). Although 5-HT also relaxes chromatophore muscles (Messenger, 2001), blocking 5-HT with methysergide at lower concentrations did not result in continuously expanded chromatophores (10%; Figure 3J; n = 3) but maintained papillae expression.

**Figure 4:**
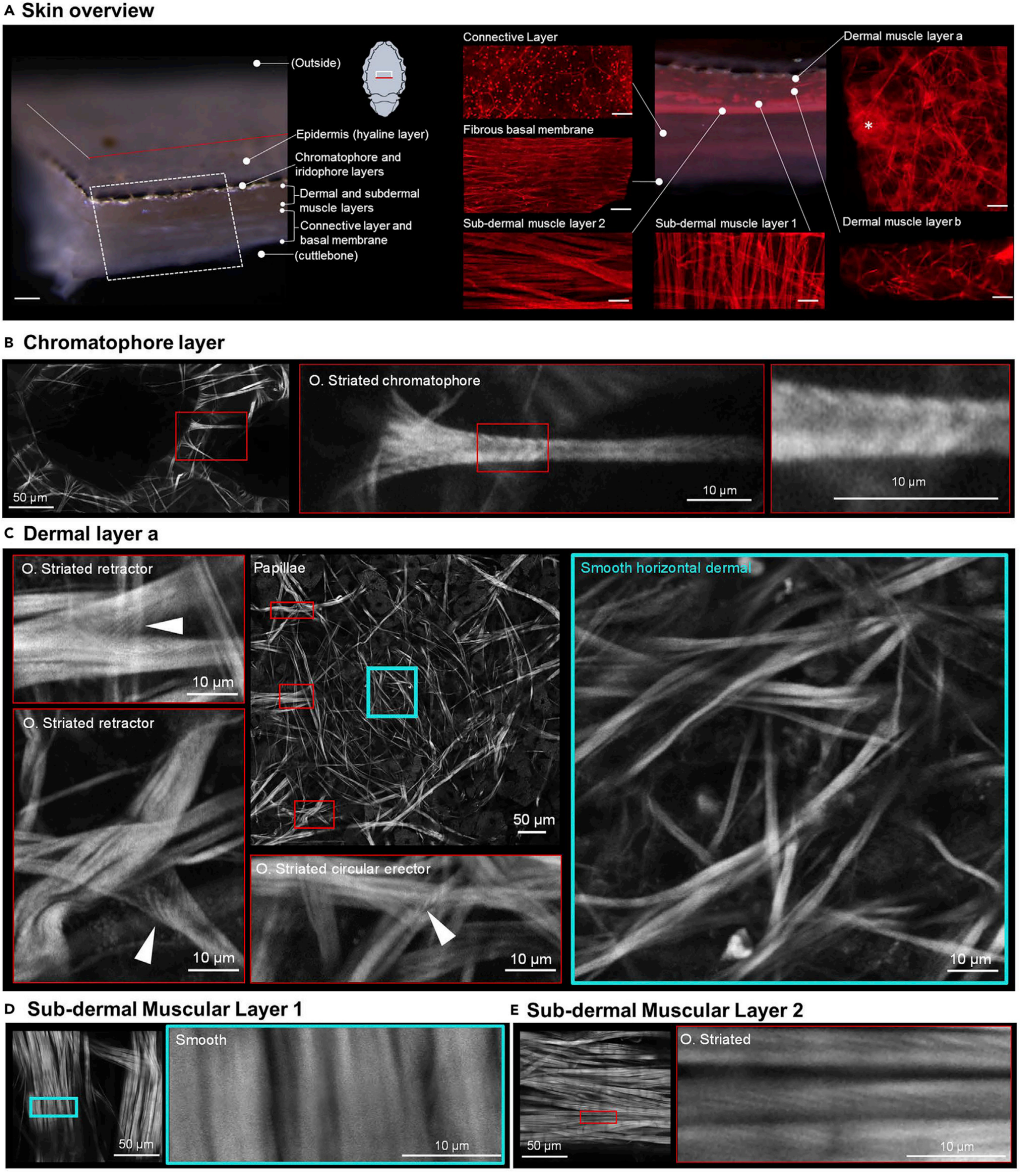
F-Actin Labeling of Skin Muscle Layers with Phalloidin (A) Skin overview. Cuttlefish skin is composed of several layers. Muscles are found in the muscular dermal layer and the two sub-dermal layers. Red labeling is phalloidin-DyLight554 stain. The pictures of the connective layer and basal membrane were taken with longer exposure to detect their structure from autofluorescence. All scale bars, 200 µm. *, papilla. (B) Phalloidin labeling of obliquely striated chromatophore muscles. Squares indicate areas shown at higher magnification. (C) Phalloidin labeling of the dermal muscular layer a shows a mix of obliquely striated (retractor and circular erector papillary muscles; red squares) and smooth (horizontal muscles of papillary core; cyan square) muscles. Arrows indicate striations on muscles. (D) Sub-dermal layer 1 is composed of smooth fibers running along the longitudinal axis of the animal. (E) Sub-dermal muscular layer 2 is composed of obliquely striated fibers running across the animal. See also Figure S2 and Movie S4.

The neurotransmitter/neuromodulator results were consistent with the substances expected to induce and release a catch-like state but do not provide a definitive answer because muscle structure is fundamental for catch. For example, mollusk catch muscles are smooth (Twarog, 1967c). Although chromatophore muscles are sensitive to acetylcholine, FMRFamide, and serotonin, they are also striated and do not display a catch-like phenotype. In chromatophores, FMRFamide produces a slower expansion and retraction dynamic than L-glutamate and each transmitter acts independently (Loi and Tublitz, 2000). It is known that cuttlefish have both striated and smooth-like muscles in their two tentacles, and the mix is thought to provide fast tentacle actuation but help maintain them coiled when not in use (Grimaldi et al., 2004). To elucidate if cuttlefish skin has a mix of smooth and striated muscles, we applied phalloidin, an f-actin label. After labeling, the different skin layers became clearly visible, which allowed us to separate them manually (Figure 4A) and inspect them with high detail with a two-photon microscope. As expected from previous reports, the phalloidin label showed oblique striations in the chromatophore muscles (Bell et al., 2013) as well as the papillary retractor and circular erector muscles (Allen et al., 2014) (Figures 4B and 4C). However, within the same optical slices (in sub-dermal layer a), we found that the internal volume of the papillae (referred to as papillae core) was made of smooth horizontal dermal muscles (Figure 4C). Although it is true that some striations may be difficult to see owing to muscle orientation, the lack of striations in horizontal dermal muscles crossing the papillae and their presence in the retractors/circular erectors were consistent in all the preparations that we imaged (n = 3). Similarly and consistently across preparations, we found that the muscles of the sub-dermal layer 1 are smooth, but those of sub-dermal layer 2 are striated (Figures 4D and 4E).

Our denervation study points toward a catch-like system for maintenance of papillae form. We have also carried out electrophysiological experiments whereby we stimulated fascicles of dermal nerves (See Transparent Methods), which demonstrated the presence of a fast system (<1 s) for papillae expression and retraction (Figures S2E and S2F, Movie S4). It is noteworthy that stimulating the papillae for longer than 30 s leaves them partially expressed for many minutes. This lingering expression is also similar to the bivalve catch muscle, where the sustained contraction (catch mode) can be elicited with DC stimulation (Twarog, 1967b, Twarog, 1967a, Twarog, 1967c). The application of neuroactive substances is consistent with both the catch process of bivalves and non-catch muscle of cephalopods, and the microscopy results show that the cuttlefish skin is composed of several muscular layers, with both striated and smooth muscles.

## Discussion

Octopus and cuttlefish can neurally control the dynamic morphing of their skin from flat 2D (i.e., no visible bump) to physical 3D texture in different grades of expression. Individual papillae in the living cuttlefish can be fully expanded (or retracted) in less than 1 s (Panetta et al., 2017). This ability allows these benthic animals to be cryptic on substrates or masquerade as nearby objects, such as kelp, algae, or coral (Panetta et al., 2017). Each species has a fixed repertoire of papillae shapes, and the biomechanical mechanism of action is that of a muscular hydrostat (Allen et al., 2013). Here we investigated the neural basis of controlling the different muscles in this unique “hydrostatic system” and found that (1) papillae signals targeting the mantle are routed through the stellate ganglion, (2) motoneurons that control the majority of the mantle papillae originate in the stellate ganglion and exit this peripheral relay center via the fin connective (made up of approximately five nerves) to re-join the fin nerve, and (3) this connective carries neural activity that also controls overall skin tension but not chromatophore excitation. By backfilling this connective, we showed three neuron populations. The majority of the fibers labeled formed population 1, with their cell bodies located in the ventral medial wall of the stellate ganglion. Population 2 consists of a small number of cell bodies in the dorsal-medial wall. Because the dorsal wall hosts sensory neurons, these are likely pressure receptors from the skin (e.g., Young, 1972). Population 3 is made of a small number of fibers that continue through into the pallial nerve (also found in squid [Gonzalez-Bellido et al., 2014]). We argue that the papillary motoneurons are those of population 1 for the following reason: it is well known that if following animal death one does not remove the connective sheaths surrounding the ganglia quickly to expose the ganglion to oxygenated water, the synapses within it start to fail (Choi et al., 2014, Colton et al., 1992), with the consequent lack of motor output even when the pallial nerve is strongly stimulated. This is known to be the case for the giant axon system and the iridescence system in squids (Gonzalez-Bellido et al., 2014). We found this to also be the case with papillae: stimulation of the pallial nerve causes papillary expression straight after dissection but fails to do so shortly after (∼10 min), whereas chromatophore expression due to pallial nerve stimulation continues reliably for hours (Figures S2A–S2D).

Hence, the mantle papillae motoneurons in cuttlefish and the mantle iridescence motoneurons in squid appear to follow homologous pathways (see Gonzalez-Bellido et al., 2014) and share a similar location for cell bodies in the stellate ganglion. In the mantle of the two species studied these systems are mutually exclusive; *Sepia officinalis* cuttlefish have papillary control without tunable iridescence (acetylcholine application or electrical stimulation has no observable effect on their iridescence; data not shown), whereas *Doryteuthis pealeii* squid have neurally tunable iridescence (Wardill et al., 2012) but lack papillae (Hanlon, 1982). Therefore, an evolutionary question arises. It is parsimonious to propose that the two systems diverged from a common ancestor that already possessed a peripheral nervous system for controlling skin tension. Alternatively, did cuttlefish gain skin texture control by hijacking an existing iridescence control circuit? This second explanation is also plausible because squids are thought to be the basal coleoid group (Sutton et al., 2016). This question could be answered by studying papillae versus iridescence control in key coleoid species. For now, the results presented here advance our knowledge about the control circuit for these two skin elements and provide a novel framework in which to investigate the neural circuitry and brain areas that control them.

Our accidental finding that papillae expression can be maintained in the absence of descending neural input led us to investigate whether it is feasible that some papillae muscles could operate in a catch-like state. Catch was originally described in bivalves, where it allows the anterior byssus retractor muscles to sustain a contraction that resists stretch without the need for continuous stimulation from motoneurons (Siegman et al., 1998). The catch mechanism of bivalves is well studied; a giant thick filament, known as twitchin, binds to thin filaments when unphosphorylated, thereby completely bypassing any actin-myosin cycling (Funabara et al., 2007). Catch allows posterior *Mytilus* adductor muscles to keep their bivalve shell firmly closed with a state of low energy consumption (Galler et al., 2010). In this regard, the presence of a catch-like mechanism in papillae would be highly beneficial, because papillae are muscular hydrostats in continuous use (Allen et al., 2009, Allen et al., 2010, Allen et al., 2014) and thus must incur considerable energy expense. The micro-muscles that form the papillae are too small and intricate to be isolated and permeated for experimental tests as undertaken with bivalve muscles. Thus, to test whether the catch-like state of the papillae is likely to follow the same mechanism as the catch of bivalve abductors, we first focused on the active compounds that control them. Acetylcholine has been previously demonstrated to excite bivalve catch muscles (Ishii et al., 1986, Hirata et al., 1989, Siegman et al., 1998), and FMRFamide has been demonstrated to induce contraction in the anterior byssus retractor muscle, a catch muscle of *Mytilus* (Hirata et al., 1989). It is therefore exciting that we found papillary muscles to be very sensitive to FMRFamide and Acetylcholine, in addition to L-glutamate, which drives a fast skin response. In addition, papillae seem to share the same sensitivity to FMRFamide that bivalve catch muscles do, as FMRFamide contracture is 3- to 30-fold lower threshold than acetylcholine (Painter, 1982). In bivalves, although FMRFamide seems to be more potent, it elicits less forceful contractions. We also found that the effect of FMRFamide and acetylcholine on papillae contraction was not identical. For example, only acetylcholine produces papillae “white-caps” in cuttlefish (i.e., forms the leucophore layer into a sphere). It is possible that the white-cap phenotype here reported results from activation of the specialized circular muscles located at the base of each leucophore mass (Allen et al., 2014). Interestingly, acetylcholine is the only compound that elicits iridescence in squid skin (Mäthger et al., 2004, Cooper et al., 1990, Hanlon et al., 1990), and Wardill et al. (2012) reported that the iridescent splotches in squid (called iridophores) are nested on a set of micro-muscles whose role is yet to be determined. It will also be interesting to know if these iridophore muscles also display catch-like properties and whether they have any effect on persistence of the iridescence signal when electrically stimulated.

We also investigated the release mechanism for the catch-like state. In bivalves, once twitchin is phosphorylated by serotonin, the catch is released and relaxation occurs (Siegman et al., 1998, Hooper et al., 2008). Our results are consistent as we showed that (1) 5-HT relaxes the papillae completely and (2) inhibiting 5-HT action through the injection of methysergide (Ono et al., 1987) resulted in papillae that remain expressed in the catch-like state for several hours, something not observed in chromatophores. Taken together, the evidence shows that the main neurotransmitters of the bivalve catch muscle also control papillae expression. The presence of a tension-sustaining mechanism in cuttlefish skin is supported by the f-actin labeling, which demonstrated that the papillary core and its sub-dermal base contain smooth muscles. However, a catch-like system composed of smooth muscle cannot be the only mechanism of papillae expression because (1) L-glutamate, the fast-action chromatophore excitatory neurotransmitter (Di Cosmo et al., 2006), also has some effect on papillae expression and (2) papillary retractors and circular erectors are striated muscles. We propose that in cuttlefish these muscles are activated by L-glutamate, allowing the animal to immediately express and retract the papillae. Indeed, by stimulating fascicles of nerves that were within the basal layers of the skin, we were able to rapidly express and retract papillae (<1 s, Movie S4).

An interpretation of our results is that the striated muscles are ideally located to implement the fast expression/retraction (as previously proposed [Allen et al., 2014]), whereas the smooth muscles are ideally positioned to maintain the papillae shape (base and core), and are responsible for the catch-like phenotype observed. To determine if the catch mechanism of bivalves and the catch-like properties here observed in cuttlefish skin share a mechanism, future experiments could employ calcium imaging to investigate the Ca^2+^ dynamics during the catch-like state. To finally confirm if such smooth types are in nature the same as the classic catch of bivalves, it would be necessary to label them against the twitchin isoform containing the D1 domain that is specifically expressed in catch muscles (Kusaka et al., 2008). However, it is possible that the catch-like phenotype observed here in a cephalopod is driven by mechanisms other than the D1 domain of twitchin. For example, even the twitchin from striated scallop muscles exhibits some level of “catchability” (defined as the ability to bind thick filaments tightly to thin filaments), and simply increasing its expression level could lead to catch (Tsutsui et al., 2007). In addition, smooth catch muscles also have a catch-specific isoform of myosin (Shaffer and Kier, 2012). Moreover, cuttlefish may have evolved a novel mechanism to hold the tension for prolonged periods. For example, nitric oxide has been implicated in developing tension in chromatophore muscles with a much slower time course than glutamate on its own (Mattiello et al., 2010).

Papillary catch muscles are an interesting example of how evolution has adapted the combined use of smooth and striated muscles in different taxonomic classes (bivalves lock their shells closed versus cephalopod express skin elements for camouflage and communication). Much is left to be discovered about papillary control. Understanding the interplay between the fast- and slow-acting mechanisms could provide further bio-inspiration for engineering an industrial dynamic material (e.g., Pikul et al., 2017). In summary, we propose that, in addition to a fast system for expression and retraction of papillae, coleoid cephalopods have smooth dermal muscles with catch-like properties, which could provide an energy-efficient means of maintaining papillae expression for long periods of camouflage, and that the motoneurons for papillae and iridescence take the same nerve pathways, with cell bodies located in similar stellate ganglion regions and therefore likely evolved from a common coleoid ancestor.

## Methods

All methods can be found in the accompanying Transparent Methods supplemental file.

## Supplemental Information

Document S1. Transparent Methods, Figures S1–S3, and Table S1

Movie S1. Behavioral Phenotypes of Cuttlefish with Different Denervation Treatment, Including Papillae in a Catch-like State, Related to Figure 1 (A) Fin nerve severed on the left side of the animal results in chromatophore inhibition in the posterior two regions of the mantle but full papillary expression ipsilateral to the cut (Figure 1F), as demonstrated by the fast papillae retraction during startle behavior. Note this Movie is 10 times slower than real time. (B) Animal with a left stellate connective cut and missing papillae expression (related to Figure 1G). (C) Animal with a left stellate connective cut displaying papillae in a catch-like state for over 1 hr and chromatophores silenced in part of mantle, ipsilateral to the cut. The second part of this movie demonstrates that papillae are not retracted in response to looming object (hand) and that manually pressing the papillae in the catch-like state only temporarily suppresses their shape as they return to normal within seconds (related to Figure 1I). (D) Animal with a left fin connective cut, unable to express papillae in two-thirds of the side ipsilateral to the surgery, but otherwise normal. Nerve cutting does not affect phenotypic expression on the contralateral side of the animal.

Movie S2. Bunching and Papillae Expression Caused by Electrical Stimulation of the Neurons that Form the Fin Connective, whose Cell Bodies Are Located in the Stellate Ganglion, Related to Figure 2 (A) Skin buckling and papillae expression caused by electrical stimulation of the fin connective nerves, through suction electrode. (B) Microscopy stack of a stellate ganglion with the most ventral nerve of the fin connective backfilled (red = dye; green = autofluorescence). Imaging direction is from ventral to dorsal surface of the ganglion.

Movie S3. Methysergide Injection Prevents Papillary Retraction Locally, Related to Figures 3 and S3 (A) Methysergide injected sub-dermally (10% dilution of 28.29 mM) prevented papillary retraction after injection *in vivo (adjacent to arrows)*, whereas behavior and chromatophores remained unaffected.

Movie S4. Fast Papillae Expression and Retraction Elicited through Electrical Stimulation of Different Dermal Nerve Fascicles, Related to Figures 4 and S2 (A) Electrical stimulation of fascicle of a dermal nerve results in fast papillae retraction, and when stimulating a different fascicle results in fast papillae expression.
