# Neural Control of Dynamic 3-Dimensional Skin Papillae for Cuttlefish Camouflage

**DOI:** 10.1016/j.isci.2018.03.021

**Published:** 2018-04-12

**Authors:** Paloma T. Gonzalez-Bellido, Alexia T. Scaros, Roger T. Hanlon, Trevor J. Wardill

## Main Text

(iScience *1*, 24–34; March 23, 2018)

In the originally published version of this article, the Received and Revised dates were incorrect. The correct Received date is June 21, 2017, and the correct Revised date is January 10, 2018. This has now been corrected in the article online. Our editorial team apologizes for any confusion this error may have caused.

