# Supplementary material for: Neural Control of Dynamic 3-Dimensional Skin Papillae for Cuttlefish Camouflage
Source: iScience. 2018 Jan 31;1:24–34. doi: 10.1016/j.isci.2018.01.001 (PMC6059360; doi:10.1016/j.isci.2018.01.001)
Supplement: Document S1. Transparent Methods, Figures S1–S3, and Table S1 [file mmc1.pdf]

ISCI, Volume *1*

Supplemental Information

Neural Control of Dynamic 3-Dimensional Skin Papillae for Cuttlefish Camouflage

Paloma T. Gonzalez-Bellido, Alexia T. Scaros, Roger T. Hanlon, and Trevor J. Wardill

Supplemental Information

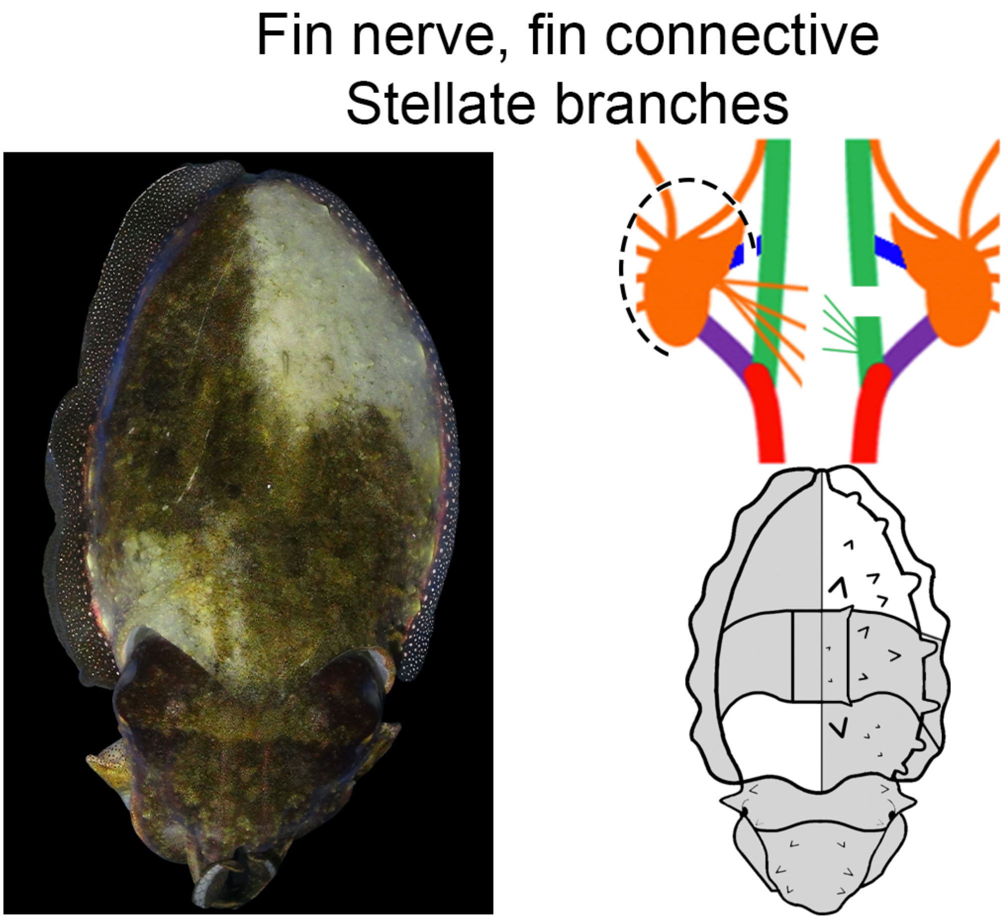

Figure S1. Additional nerves severed and their observed phenotypes, Related to Figure 1. Fin nerve severed on the animal's left side (right side of the image) results in chromatophore inhibition in the posterior two regions of the mantle, but full papillary expression ipsilateral to the cut (see equivalent response in Figure 1F). Note that the middle mantle skin region, lateral to the white square could be innervated by ether the small stellar nerves, medial to the stellate (indicated by small orange lines, potentially uncut in this animal) and/or innervated via small branches leaving the fin nerve, diagrammatically represented as small green nerves branching from fin nerve stump. The fin connective and the stellate branches severed on the animal's right side (left side of the image) abolishes chromatophore expression in the anterior region and complete inhibition of papillae expression (see equivalent response in Figure 1H).

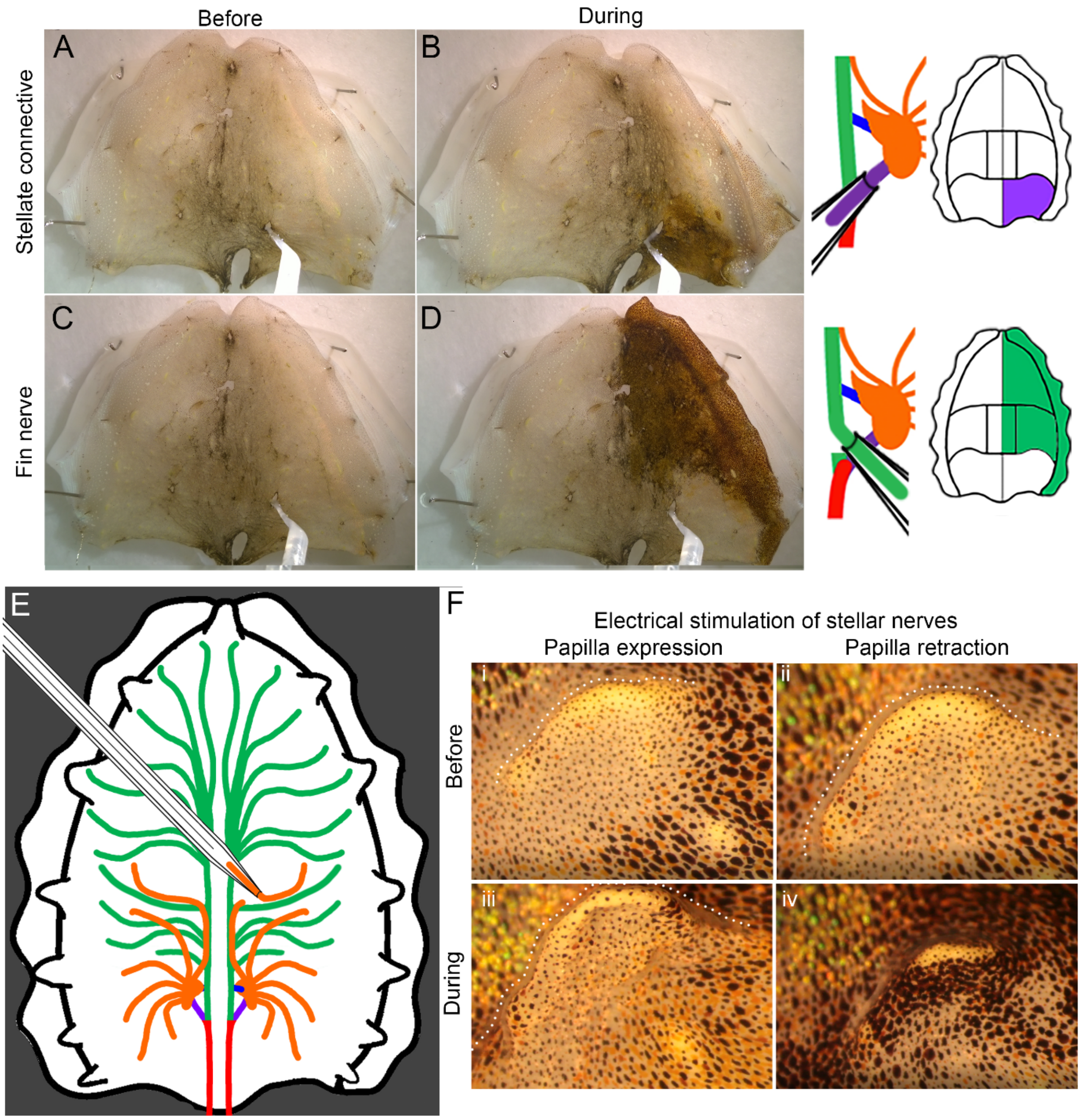

Figure S2. Electrophysiology activating expression of the chromatophores and papillae, Related to Figure 2 & 4. (A) *Ex vivo* mantle preparation before stimulation. (B) Biphasic electrical stimulation delivered for 6 seconds with 5V 50 Hz 600 µs pulses through the stellate connective causes chromatophore expansion in the anterior region, in addition to general skin bunching and small papillae expression (as evidenced by browning of the tissue, a result of increased chromatophore density caused by the skin bunching). (C) *Ex vivo* mantle preparation before stimulation. (D) Biphasic electrical stimulation was delivered for 6 seconds with 5V 10 Hz 600 µs pulses through the fin nerve causes chromatophore expansion in posterior regions and fin. (E) Diagrammatic representation of the suction electrode location on a fascicle of a dermal nerve (orange). (F) Papilla expression (i) before electrical stimulation and (ii) during electrical stimulation. Papilla expression driven by stimulation of a different fascicle of the same dermal nerve (iii) before electrical stimulation and (iv) papilla retraction during electrical stimulation. Note that chromatophores were also activated because dermal nerves also carry chromatophore motoneurons.

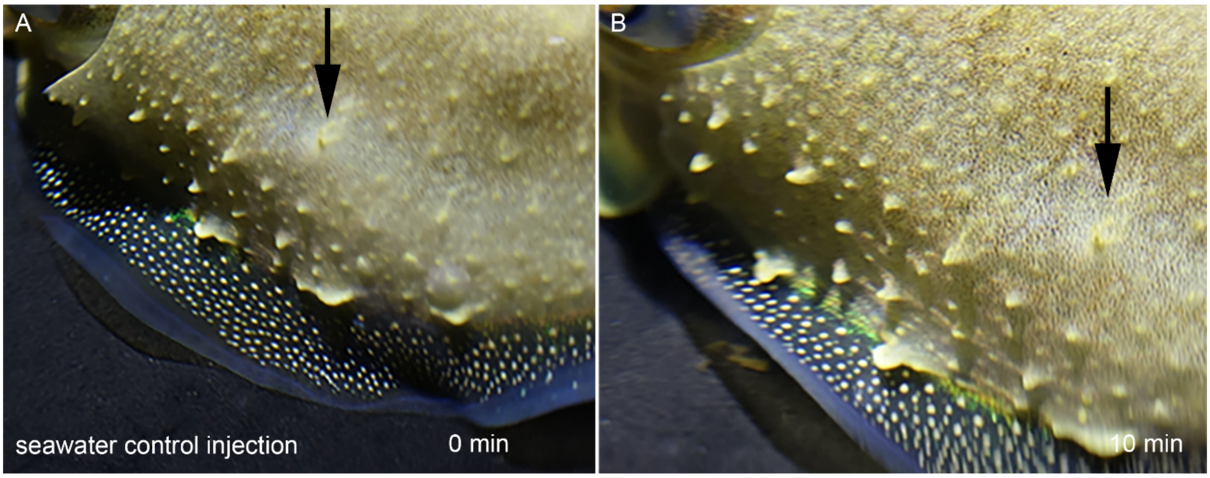

Figure S3. Seawater injections did not activate papillae, Related to Figure 3. Control injection of seawater *in vivo* did not result in any change in behavior, chromatophore, or papillary expression.

Supplementary Table

Table S1. Determining optimal neurotransmitter concentration for pharmacological injections, Related to Figure 3.

ex vivo **(mantle tissue)**	**10 µM**	**100 µM**	**1 mM**	**10 mM**
L-Glutamate	No chromatophore change<br>No papillae expression<br>No/minor skin bunching, n=4	Minor chromatophore activity<br>Minor skin buckling<br>No papillae expression<br>n=1	Minor chromatophore activity<br>Small dorsal papillae expression<br>Major skin bunching, n=2	Minor chromatophore activity<br>Possible papillae expression masked by major skin bunching<br>n=4
Acetylcholine	Chromatophore activity<br>Papillae expression<br>White caps<br>No skin bunching, n=1	Chromatophore activity<br>Papillae expression<br>White caps<br>Minor skin bunching, n=2	Minor chromatophore activity<br>(*probably injected in a skin layer too deep)<br>n=1	Chromatophore activity<br>Papillae expression<br>White caps<br>Minor skin bunching, n=5
FMRFamide	No chromatophore change<br>No papillae expression<br>Minor skin bunching, not sustained<br>n=1	No chromatophore change<br>Small and medium sized papillae expression, noticeable just before major skin bunching<br>n=9	N/A	N/A
in vivo	**10 µM**	**100 µM**	**1 mM**	**10 mM**
FMRFamide	N/A	N/A	N/A	Dark chromatophore<br>Papillae expressed<br>Skin bunching<br>n=3
L-Glutamate	N/A	N/A	N/A	Dark chromatophore<br>Small dorsal papillae expression<br>Skin bunching<br>n=3
Acetylcholine	N/A	N/A	N/A	Dark chromatophore<br>Papillary expression<br>White caps<br>n=6
Serotonin	N/A	N/A	Papillary inhibition; chromatophore inhibition<br>n=4	N/A
**Methysergide**	**10ng/mL (100%)**	**50%**	**10%**	**1%**
in vivo	Constantly expressed chromatophores<br>Papillae large<br>n=2	Constantly expressed chromatophores Papillae expressed<br>n=3	No change in chromatophores<br>Excited papillae<br>n=2	No change in chromatophores<br>Excited papillae<br>n=5
ex vivo	Chromatophore inhibition<br>Small dorsal papillae<br>No skin bunching<br>n=1	N/A	N/A	N/A

Transparent Methods

Key Resources Table

REAGENT or RESOURCE	SOURCE	IDENTIFIER
Chemicals, Peptides, and Recombinant Proteins		
L-glutamic acid monosodium salt monohydrate	Sigma Aldrich	G1626
FMRFamide (Phe-Met-Arg-Phe)	Sigma Aldrich	P4898
Acetylcholine chloride	Sigma Aldrich	A6625
Serotonin	Sigma Aldrich	14927
Methysergide maleate salt	Sigma Aldrich	M137
Dimethyl sulfoxide	Sigma Aldrich	D2650
Phalloidin-DyLight554	New England Biolabs	13054S
Phalloidin-DyLight633	ThermoFisher	21840
Lucifer yellow	Invitrogen	L-453
Collagenase	Roche	10269638001
Hyaluronidase	Sigma-Aldrich	H4272
NeutrAvidin conjugated to DyLight 633	Thermo Scientific	22844
anti-lucifer yellow antibody (rabbit IgG fraction; biotin-XX conjugate)	Molecular Probes	A-5751
2,2′-thiodiethanol (TDE)	Sigma-Aldrich	88559
Experimental Models: Organisms/Strains		
Sepia officinalis	MBL	Eggs collected from southern England, juveniles and adults cultured at MBL
Doryteuthis pealeii	MBL	Wild caught
Software and Algorithms		
Adobe Photoshop CC 2015	Adobe Systems, Inc.	http://www.adobe.com/products/photoshop.html
Microsoft PowerPoint 2010	Microsoft Corp.	https://products.office.com/en-us/microsoft-powerpoint-2010
FIJI ImageJ v3.2.1	National Institute of Health	Schindelin et al., 2012
Vaa3D v3.1	Peng et al. 2010	http://www.alleninstitute.org/what-we-do/brain-science/research/products-tools/vaa3d/

Further information and requests for resources and reagents should be directed to and will be fulfilled by the Lead Contact, Trevor Wardill (tjw79@cam.ac.uk).

Experimental model details

Sepia officinalis

Juvenile cuttlefish (*Sepia officinalis,* n=18) were cultured and maintained at the Marine Resources Center of the Marine Biological Laboratory in Woods Hole, Massachusetts, USA (Panetta et al., 2017). The sex of specimens was not determined as skin elements studied here are not known to be sexually dimorphic. Animals used were between 6 and 18 months old. For electrophysiology and pharmacology experiments, older animals (12-18 months old) were used for ethical reasons, as they had already been used in behavioral experiments in the laboratory and would die in the following year. Younger animals (6 months) were used for nerve backfilling, which was necessary for the tissue to be small enough to allow volumetric imaging. All animal procedures were in compliance with ARRIVE guidelines regulating animal experimentation. All efforts were made to minimize animal suffering and to reduce the number of animals used. To minimize stress, *Sepia officinalis* were held at low density (1–3 cuttlefish) in each holding pen, made using divisions within large tanks. The tanks had a continuous high flow recirculating supply of seawater at 15±0.5°C. The cuttlefish were fed twice daily as juveniles with various live food and as adults with defrosted frozen shrimp. Only the animals that had fed well for at least 1 day, a sign of strength and drop in stress levels, were used for surgical experiments. For animal transfer, cuttlefish were caught with a bowl and carefully moved between enclosures. Any signs of stress displayed by the cuttlefish did not last more than a few seconds. Individuals were allocated randomly to multiple experimental groups to reduce number of animals used, in the case of pharmacological injection and papillae behavior experiments when such overlap did not result in an interaction and there were several days for the individuals to rest between experiments. Denervated animals (n=9) also participated in papillae expression behavioral experiments and pharmacological injections.

Doryteuthis pealeii

Adult Atlantic longfin squid (*D. pealeii*, aka. *Loligo pealeii;* n=2) were collected by brief trawling runs from the coastal waters near Woods Hole, Massachusetts. Animal housing and maintenance was previously reported (Wardill et al., 2012). The smallest available animals (15 cm long) were chosen, and the two ganglia used for the nerve backfills.

Experimental methods

Surgery

For the experiments involving a fast denervation, the animals were lightly anesthetized by submersion in 1.5% EtOH in seawater for 30 s or until unresponsive, before being held by hand for a fast nerve section (< 5 seconds). For the experiments that required extensive *in vivo* dissection to expose nerves, the animals were first deeply anesthetized with 3% EtOH for 30 seconds or until unresponsive, then placed in a recirculating oxygenated and Tris-buffered sea water bath (18°C) with 1.5% EtOH (with jets directed at their gills), for the duration of the surgery. Using small surgical scissors, the connective sheath was removed to expose the nerve of interest which was then severed with no harm to the surrounding nerves or tissue. For *ex*

vivo experiments, the animals were anesthetized by submersion in 3% EtOH until unresponsive, followed by decapitation and decerebration. All procedures carried out in this study comply with institutional recommendations for cephalopods. After surgery, all live animals were placed in recirculating cold-water tanks (18°C). Straight after surgery the animals were monitored for any sign of stress and photographed for effect on papillary expression, then left to heal for several days before pharmacological testing was conducted. The *ex vivo* tissues were quickly placed in Tris-buffered sea water bath (18°C) for experimental procedures.

Stimulation experiments

For *ex vivo* experiments, the mantle was fully opened and pinned to allow for full visualization of both stellate ganglia. Electrodes were placed on the ends of the cut nerve, and suction was applied so that several millimeters of nerve reached into the electrode. Electrophysiological settings were varied depending upon experimental setup, but most consisted of 5V (or 50V), 10-50 Hz biphasic electrical stimulation of 600 µs pulses for a varying amount of time (6-50 seconds). Videography was conducted as previously reported (Wardill et al., 2012). Once a nerve was connected to the stimulator (model 2100, A-M Systems), we coordinated electrical stimulation with videography (through the trinocular port of the stereo microscope), using a Power Lab data acquisition unit (PL3504) and Lab Chart software (AD Instruments) to trigger equipment, including a blue light-emitting diode (LED) light pulse (observable in the video or spectra measurements). High-definition video was collected at 30 frames per second with a Canon EOS 5D Mark II digital camera in manual mode.

Papillae expression observations

Cuttlefish were tested in an experimental chamber supplied with continually running seawater and surrounded by dark curtains to shield the animals from outside stimuli (Boal and Ni, 1996, King and Adamo, 2006, Allen et al., 2009). Cuttlefish were placed on black felt substrate inside a circular arena 24.5 cm in diameter and 8.0 cm tall. The experimental chamber was illuminated with a circular 40 W Fluorescent light source (Phillips CoolWhite; illumination at the level of the arena was approximately 1,000 lux). Before a trial began, each animal was allowed to acclimate to the experimental chamber. A cuttlefish was considered “settled” when it was sitting still on the substrate, maintaining a stable body pattern. This occurred within 5–20 min from introduction to the experimental chamber. Following acclimation, a five-megapixel digital photograph was taken remotely with a Nikon Coolpix 5400 camera, as per Allen et al. (2009) for a pre-experimental control.

Pharmacological experiments

For a control, buffered sea water of the same pH, temperature, and salinity of the environment was injected subcutaneously at the same depth and angle as the chemicals. No papillary reaction occurred (see **Figure S3**). FMRFamide (Sigma Aldrich 4898) acetylcholine (Sigma Aldrich A6625), L-Glutamate (Sigma Aldrich, 49621), serotonin Sigma Aldrich, 14927, 1 mM), Methysergide (Sigma Aldrich M137, 28.29 mM in DMSO) were injected at various concentrations. Because methysergide was dissolved in dimethyl sulfoxide (DMSO), an *in vivo*

100% DMSO injection control performed. This showed that DMSO levels injected did not interfere with normal papillary function (ability to be expressed and relaxed, data not shown).

Phalloidin muscle histochemistry

Approximately 2 x 2 mm pieces of skin, previously fixed with 4% PFA for 12 hours, were cut with micro-scissors, then immersed in phalloidin-DyLight554 dissolved in methanol (New England Biolabs #13054S; 300 units ml–1) for 3 minutes at 25°C in the dark. The pieces where then picked up with tweezers and actively washed in PBS for 1 minute to remove unbound dye (PBS, 1x Phosphate buffer saline, Oxoid tablets # br0014g). This treatment rendered the muscle layers visible by fluorescence along the edges of the tissue. Under fluorescence light, viewing tissue through a stereomicroscope (Leica M165FC), layers were separated manually (using micro-scissors), according to their specific labelling patterns. The different layers were subsequently immersed in phalloidin-DyLight633 dissolved in methanol (ThermoFisher #21840; 300 units ml–1) for 3 - 5 mins. Tissue was then washed in PBS as above. A large drop of 80% glycerol (Fisher Scientific #G/0650/17) in PBS was placed on a cover slip (VWR, 22 x 40 mm, # 630-1590), previously attached by nail varnish to a custom slide (with a 17 mm hole where the sample can be viewed from either side). The tissue was transferred to the glycerol drop and swirled around to encourage mixing. Another coverslip was placed on top, and a large bullet shaped fishing sinker used to hold the tissue flat and in place. Nail varnish was then used to seal the edges. The mounted samples were imaged with a Zeiss 25×, 0.8 NA, objective (Carl Zeiss 440852-9870-000; 600 µm working distance) corrected between oil and glycerol, a Newport Spectra-Physics InSight® DS+™ laser at 920 nm, and a Bruker (Prairie Technologies) *in vivo* 2-photon microscope using GFP (500-540 nm emission) and RFP (610-650 nm emission) detection channels for detection of phalloidin-DyLight633. Minimal fluorescence from DyLight554 was detected by our 2-photon filter sets with 920 excitation, but DyLight554 could be viewed with our fluorescence ET RFP long pass filter sets.

Fin connective back-fills with Lucifer yellow

We back-filled the largest nerve that forms part of the fin connective with 3% Lucifer yellow (LY; Invitrogen, #L-453) using a similar procedure to Isaacson and Hedwig (Isaacson and Hedwig, 2017). The largest nerve of the fin connective was located, and supported with a metal spoon (bent needle). Water level was lowered, the nerve was dried with a piece of paper and protease crystals applied for 3 minutes. The nerve was then jet washed several times, dried with a piece of filter paper and then sucked immediately into a tight-fitting glass pipette containing the 3% LY and injected with current (between 50 and 100 µAmps) for 5 to 10 minutes. In successful preparations, the dye could be seen traveling in the first minute. The stellate was then dissected out of the tissue, pinned in a dish, covered in sea water and left in the fridge overnight to allow the dye to travel further. At this point the ganglia where imaged with a fluorescence scope before being immersed in 4% paraformaldehyde in Tris-buffered sea water, then triple rinsed and stored in Tris-buffered sea water at 4°C.

Tissue was processed as described previously (Gonzalez-Bellido and Wardill, 2012). Briefly, the tissue was dehydrated and rehydrated in a series of steps to remove lipids and any trapped air, and then permeated with collagenase (Roche, 10269638001; 0.5 mg ml–1) and

hyaluronidase (Sigma-Aldrich, #H4272; 300 μg ml–1) to allow penetration. An anti-LY antibody (rabbit IgG fraction; biotin-XX conjugate; Molecular Probes, #A-5751) was used in conjunction with NeutrAvidin conjugated to DyLight 633 (Thermo Scientific, #22844) to shift the excitation into the red and away from tissue autofluorescence. Tissue was then cleared in thiodiethanol (TDE; Sigma-Aldrich, #88559) and mounted inside SYLGARD® 184 silicone elastomer chamber (inside a deep plastic container) and secured down with a cover glass. Automated imaging was undertaken with Bruker (Prairie Technologies) *in vivo* 2-photon microscope and a long-distance Olympus XLSLPLN25XGMP objective corrected for oil, a Newport Spectra-Physics InSight® DS+™ laser at 920 nm, and a Bruker (Prairie Technologies) In Vivo Microscope using GFP and RFP detection channels. Multiple tiled z-stacks were collected with brightness correction to compensate for increasing depth. Image z-stacks were stitched with the 'Grid/Collection stitching' plug-in within Fiji software, v. 1.46p (Preibisch et al., 2009). The NeurAvidin also labelled blood vessels in this tissue, which prevent us from tracing individual neurons, but different populations could be clearly seen. The stacks were viewed with freely available software Vaa3D, v. 3 (Peng et al., 2010) and Fiji.

Quantification and statistical analysis

Final figures were constructed using Adobe Photoshop CC 2015 (Adobe Systems, Inc., San Jose, CA, USA; http://www.adobe.com/products/photoshop.html) with adjustments for size, contrast, and brightness to ensure figure consistency without compromising scientific validity. Schematic diagrams were drawn using Adobe Photoshop CC 2015 and Microsoft PowerPoint 2010 (Microsoft Corp. Redmond, WA, USA).

Supplemental References

Allen, J. J., Mathger, L. M., Barbosa, A. & Hanlon, R. T. 2009. Cuttlefish use visual cues to control three-dimensional skin papillae for camouflage. *J Comp Physiol A Neuroethol Sens Neural Behav Physiol,* 195**,** 547-55.

Boal, J. G. & Ni, J. N. 1996. Ventilation rate of cuttlefish, *Sepia officinalis*, in response to visual stimuli. *Veliger,* 39**,** 342-347.

Gonzalez-Bellido, P. T. & Wardill, T. J. 2012. Labeling and confocal imaging of neurons in thick invertebrate tissue samples. *Cold Spring Harb Protoc,* 2012**,** 969-83.

Isaacson, M. D. & Hedwig, B. 2017. Electrophoresis of polar fluorescent tracers through the nerve sheath labels neuronal populations for anatomical and functional imaging. *Sci Rep,* 7**,** 40433.

King, A. J. & Adamo, S. A. 2006. The ventilatory, cardiac and behavioural responses of resting cuttlefish (*Sepia officinalis* L.) to sudden visual stimuli. *Journal of Experimental Biology,* 209**,** 1101-1111.

Panetta, D., Solomon, M., Buresch, K. & Hanlon, R. T. 2017. Small-scale rearing of cuttlefish (*Sepia officinalis*) for research purposes. *Marine and Freshwater Behaviour and Physiology,* 50**,** 115-124.

Peng, H. C., Ruan, Z. C., Long, F. H., Simpson, J. H. & Myers, E. W. 2010. V3D enables real-time 3D visualization and quantitative analysis of large-scale biological image data sets. *Nature Biotechnology,* 28**,** 348-353.

Preibisch, S., Saalfeld, S. & Tomancak, P. 2009. Globally optimal stitching of tiled 3D microscopic image acquisitions. *Bioinformatics,* 25**,** 1463-1465.

Wardill, T. J., Gonzalez-Bellido, P. T., Crook, R. J. & Hanlon, R. T. 2012. Neural control of tuneable skin iridescence in squid. *Proceedings of the Royal Society B-Biological Sciences,* 279**,** 4243-52.
